# The structure–function relationship between multifocal pupil perimetry and retinal nerve fibre layer in glaucoma

**DOI:** 10.1186/s12886-024-03402-z

**Published:** 2024-04-10

**Authors:** Corinne F. Carle, Allan Y. H. Chain, Maria Kolic, Ted Maddess

**Affiliations:** 1 Neuroscience, The John Curtin School of Medical Research, Australian National University, Building 131 Garran Road, Canberra ACT, 2601 Australia; 2 CERA Retinal Gene Therapy Unit, University of Melbourne, Melbourne Vic, Australia

**Keywords:** Objective perimetry, Glaucoma, Retinal nerve fibre layer, Structure/function

## Abstract

**Background:**

Multifocal pupillographic objective perimetry (mfPOP) is a novel method for assessing functional change in diseases like glaucoma. Previous research has suggested that, in contrast to the pretectally-mediated melanopsin response of intrinsically photosensitive retinal ganglion cells, mfPOP responses to transient onset stimuli involve the extrastriate cortex, and thus the main visual pathway. We therefore investigate the correlation between peripapillary retinal nerve fibre layer (pRNFL) thickness and glaucomatous visual field changes detected using mfPOP. Parallel analyses are undertaken using white on white standard automated perimetry (SAP) for comparison.

**Methods:**

Twenty-five glaucoma patients and 24 normal subjects were tested using SAP, 3 mfPOP variants, and optical coherence tomography (OCT). Arcuate clusters of the SAP and mfPOP deviations were weighted according to their contribution to published arcuate divisions of the retinal nerve fibre layer. Structure–function correlation coefficients (*r*) were computed between pRNFL clock-hour sector thickness measurements, and the local visual field sensitivities from both SAP and mfPOP.

**Results:**

The strongest correlation was observed in the superior-superotemporal disc sector in patients with worst eye SAP MD < -12 dB: *r* = 0.93 for the mfPOP LumBal test (*p* < 0.001). Correlations across all disc-sectors were strongest in these same patients in both SAP and mfPOP: SAP *r* = 0.54, mfPOP LumBal *r* = 0.55 (*p* < 0.001). In patients with SAP MD ≥ -6 dB in both eyes, SAP correlations across all sectors were higher than mfPOP; mfPOP correlations however, were higher than SAP in more advanced disease, and in normal subjects.

**Conclusions:**

For both methods the largest correlations with pRNFL thickness corresponded to the inferior nasal field of more severely damaged eyes. Head-to-head comparison of mfPOP and SAP showed similar structure–function relationships. This agrees with our recent reports that mfPOP primarily stimulates the cortical drive to the pupils.

**Supplementary Information:**

The online version contains supplementary material available at 10.1186/s12886-024-03402-z.

## Background

The need for a straightforward, effective, and objective perimeter for assessment of visual function has led to the development of *multifocal pupillographic objective perimetry* (mfPOP). This technique, which uses relative changes in pupil diameter to generate a functional map of visual field sensitivity, has been shown to be both sensitive and specific to dysfunction in a number of diseases affecting the visual system. These include macular degeneration [1–3], diabetic retinopathy [4–6], multiple sclerosis [7], and glaucoma [8–10]. In glaucoma, the characteristic degeneration of retinal ganglion cells (RGCs) leads to observable changes in the optic nerve head and retinal nerve fibre layer (RNFL). This loss of afferent neurons of the main visual pathway leads to corresponding changes in visual function. The association between these anatomical and perceptual changes, the so-called *structure–function* relationship, has been extensively investigated using peripapillary RNFL (pRNFL) measurements and maps of visual sensitivity obtained using standard automated perimetry (reviewed Malik et al. [11]). The amount to which localized structural changes in glaucoma are reflected by changes in mfPOP sensitivity has not yet been addressed.

A variety of different RGC classes contribute to different components of the pupillary response [12–14]. Some of these RGCs do not contribute to visual perception and so it is possible that structure function changes for mfPOP might be different to SAP. Steady-state pupillary diameters are largely mediated by retinotectally-projecting intrinsically photosensitive RGCs (ipRGCs) [14]. We have shown that the ability to detect glaucomatous scotomas is impaired using long-duration specialised mfPOP stimuli targeting the ipRGC melanopsin response, suggesting either that ipRGCs may be less involved or that their large and overlapping receptive fields reduce the spatial resolution of measurements [10]. We have recently shown that the responses to the standard transient mfPOP stimuli are mediated by the extrastriate cortex [15], as expected from the neuro-anatomy [12]. This should mean that, in glaucoma, the pattern of pupillary responses produced by these brief mfPOP onset stimuli is subject to similar factors as standard automated perimetry. Pupillary responses to mfPOP stimuli are, therefore, likely to be directly affected by the loss of RGCs in the main visual pathway. It follows then, that the topography and degree of reductions in mfPOP sensitivity should correlate with reductions in retinal nerve fibre layer thickness in glaucoma.

Published models of nerve-fibre trajectories now allow topographic mapping of pRNFL sectors to circumscribed areas of retina. One such model, by Jansonius et al. [16], will be used here to explore the structure–function relationships of mfPOP and SAP in subjects with and without glaucoma.

## Methods

### Subjects

Twenty-five subjects with open-angle glaucoma, recruited from the Canberra Eye Hospital, and 24 with healthy vision, recruited from Canberra optometric practices or by word-of-mouth, were enrolled in this study. Diagnostic status of subjects was confirmed using Stratus OCT, Humphrey Field Analyzer II (HFA) achromatic perimetry (24–2, size III, SITA-Fast) and Matrix 24–2 perimetry, (all from Carl Zeiss Meditec Inc., Dublin, CA), slit-lamp biomicroscopy and applanation tonometry. Exclusion criteria included evidence of other ocular pathology or previous ocular surgery (Argon or Selective Laser Trabeculoplasty excepted in patients), refractive errors greater than ± 6 diopters or more than 2 diopters of cylinder, or systemic disease or medication that might impair vision or pupillary responses. Subjects were requested not to consume caffeine or alcohol for one hour before testing. Glaucoma subjects were classified according to the HFA mean deviation (MD) of whichever of their eyes had the worst value: *Mild*, worst eye MD ≥ -6 dB; *Moderate*, worst eye MD < -6 dB but ≥ -12 dB; and *Severe*, worst eye MD < -12 dB.

### Multifocal infrared pupil perimetry

**Fig. 1:**
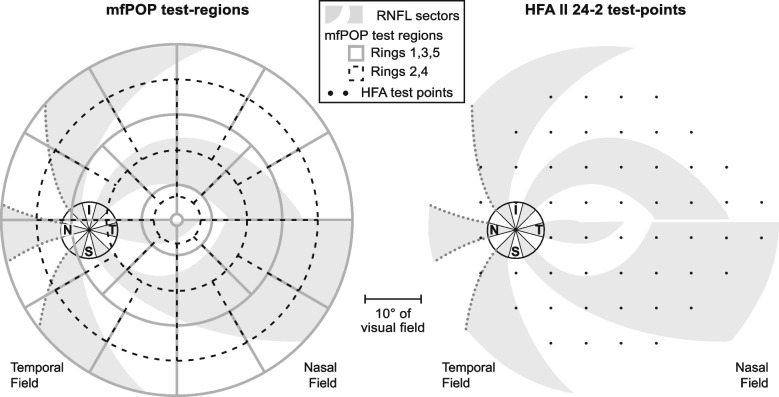
Alignment of RNFL, OCT, mfPOP and HFA. Left-eye equivalent maps of the optical coherence tomography (OCT) sectors and their retinal nerve fibre layer (RNFL) trajectories, with multifocal pupillographic objective perimetry (mfPOP) test regions (left) and Humphrey Field Analyzer (HFA) test-points (right) superimposed. Grey and white radiating arcuate patches delineate the pRNFL trajectories within the divisions which were defined using the RNFL model.(Jansonius NM et al. [16]) Grey dotted lines on pRNFL sector boundaries represent estimated trajectories in the nasal retina, as a result of the absence of RNFL data in the nasal region of the Jansonius model. Retinal location of the OCT pRNFL sectors are indicated by: S—Superior; I—inferior; N—Nasal; T—Temporal. The boundaries of the mfPOP regions as shown somewhat exaggerate the degree of overlap because stimulus edges fade to zero contrast (see e.g. Carle CF et al. [18])

The mfPOP tests were conducted using a prototype of the Konan objectiveFIELD Analyzer (OFA, Konan Medical Inc., Irvine CA), which has FDA clearance. MfPOP utilizes dichoptic presentation of spatially-sparse multifocal stimuli. The stimuli and methods used here have previously been described in detail [4, 8, 17–19]. In short, pupil diameters in both eyes are video-monitored under infrared illumination and frames during blinks are discarded. In this study, transient stimuli of 33 ms duration were presented in each of 88 test-regions (44/eye, Fig. 1) with an average presentation interval of 4 s in each test-region, thus providing around 11 stimuli per second to each eye concurrently. Responses arising from stimulation of each test-region are extracted from the raw continuous pupillary response using multiple linear regression [17]. A single test comprised eight 30 s segments separated by short breaks, giving overall test durations of 6 min for both eyes. Therefore, response estimates for each region are effectively the mean of the subject’s pupillary responses to a median of 60 stimulus presentations. The amplitudes of these responses are standardized to a baseline pupil diameter of 3500 μm which serves to reduce much of the age dependence of constriction amplitudes caused by senescent pupils [4, 8, 17, 18]. Subjects fixated a small centrally-placed cross; binocular fusion was assisted by low-contrast radial variation of the background luminance and crosshairs extending to the edge of the viewing field.

Subjects were tested using three mfPOP stimulus variants: LumBal, Lum + Col and LumBal + Col, presented in random order. LumBal consisted of luminance-balanced yellow stimuli (73–150 cd/m^2^, C.I.E. x, y coordinates 0.377, 0.464) on a yellow 10 cd/m^2^ background. Lum + Col comprised uniform-luminance green stimuli (all 150 cd/m^2^, x, y 0.291, 0.612) on a 10 cd/m^2^ red background (x, y 0.551, 0.343). LumBal + Col consisted of luminance-balanced green stimuli (65–150 cd/m^2^) on the same red background as Lum + Col. Luminance balancing involves presenting dimmer stimuli to more sensitive areas of the retina and serves to optimize the signal-to-noise ratio across all regions [4, 8]. The three mfPOP variants in this experiment differed in their relative proportion of luminance and color signal: LumBal + Col has the lowest overall luminance-contrast and therefore the highest proportion of color signal, Lum + Col has higher contrast and therefore more luminance signal, and LumBal uses luminance signal only. This allowed assessment of differences in structure–function relationships between these types of visual input to the pupil system. Earlier publications provide detailed descriptions of mfPOP methods and luminance-balancing [8, 9, 17, 20].

### Structure–function analysis

Structure–function correlations were estimated between: OCT circular peripapillary scans (Stratus OCT Fast RNFL 3.4, software version 4.0.7), and visual fields obtained using mfPOP and achromatic SAP (HFA II 24–2, size III, SITA-Fast). Custom software developed using Matlab (R2020b, The MathWorks Inc, Natick, MA) was used for all analyses. The anatomical course of RGC axons and their retinotopy as they converge on inferior, temporal, and superior clock-hour sectors of the 3.4 mm pRNFL scan was determined using Jansonius’ model of nerve fibre bundle trajectories [16]. The three nasal sectors were approximated from an earlier topographic study [21] (Fig. 1). This retinal map was then flipped along vertical and horizontal axes and overlaid on mfPOP test-region and HFA test-point maps i.e. temporal visual field on nasal retina, inferior visual field on superior retina.

The experimental measures comprised mfPOP sensitivities (mean of direct and consensual responses for each stimulus region), HFA threshold sensitivities for SAP, and pRNFL thickness for OCT. To maintain parity between mfPOP, SAP, and OCT the same method was used to estimate deviation scores for each test. This involved dividing the abovementioned values for each test-region, point and sector by the overall median of all normal subjects’ values for that particular test-region, point or sector. Congruence between OCT sectors and visual-field deviations was achieved in the following manner:

i) the relative proportion of each test-region/point falling within the arcuate division of retina corresponding to each sector was estimated using the overlaid visual-field/pRNFL maps; at this stage values for each *test-region/point* sum to 1 (Supplementary Fig. S1, Supplementary Tables S1 and S2).
ii) values within each sector were then divided by the sum of all proportions for that sector, providing weightings that represent each test-region/point’s proportional contribution to each OCT sector’s arcuate division; values for each *sector* now sum to 1.
iii) deviations from each mfPOP test-region and HFA test-point were multiplied by these weightings and then summed by OCT sector, providing sets of 12 weighted and pooled deviation scores for each visual-field.

Correlation coefficients (*r*) and associated confidence intervals were then estimated between these pooled visual-field deviations and the OCT deviations for each subject, i.e. across both eyes for the subset of data required. Spearman rank-order correlation was used in all analyses due to the non-linear relationship between HFA and OCT measures [22]. Principal curves for scatterplots were estimated using the princurve package in R [23].

Power calculations used G*Power (U Kiel, Germany). Using published data [8] for the 3 protocols we calculated the standardised effect size as Cohen’s d corrected for small sample sizes. As in that study we used the mean of the worst 6 locations in each field. We compared normal control fields and glaucoma fields whose HFA MD <  = -12. For LumBal, Lum + Col, and LumBal + Col the effect sizes were 1.48, 2.06, and 1.64 (Very Large to Huge). For d = 1.48, a *p*-value of 0.05, and a power of 0.95 the per group sample size was 13.

## Results

Of the 25 glaucoma subjects, 10 were classified as Mild (both eyes SAP MD ≥ -6 dB), 6 as Moderate (worst eye MD < -6 dB but ≥ -12 dB), and 9 as Severe (worst eye MD < -12 dB). No subject had MD < -12 dB in both eyes (Table 1). Median HFA MD, PSD and inter-eye differences for each of the Normal and Glaucoma subgroups are presented in Table 1. Because most glaucoma subjects had asymmetric visual field loss and were classified according to their worst performing eye, the median HFA MD for subjects in the Moderate and Severe glaucoma subgroups was close to the upper limits of these two classifications. The OCT pRNFL clock-hour sectors that most often fell below normal limits in glaucoma subjects were the inferior and superior sectors and those adjacent (Table 1). The median proportion of mfPOP recording lost due to e.g. blinks and fixation losses was 1.5%; the most missing from any single test was 8%.

**Table 1:** Normal and glaucoma subject characteristics

			**SAP parameters**			**OCT parameters**	
	n Subjects (males)	Mean age ± SD	Median HFA MD	Median HFA PSD	Median intereye Δ in HFA MD	Mean sectors/eye < 5th %ile	Most affected RNFL sectors^a^
**Normal**	**24 (10)**	**61.9 ± 7.3**	**-0.04**	**1.32**	**0.38**	**0.29**	**NSN, IIN, TST**
**Glaucoma** (all)	**25 (15)**	**68.0 ± 9.1**	**-4.57**	**5.71**	**3.10**	**3.72**	**I, [IIT, SST]**
Mild^b^	10 (6)	66.6 ± 11.1	-3.21	3.06	2.40	2.85	IIT, [SSN, I, SST]
Moderate^b^	6 (2)	64.8 ± 6.7	-6.29	9.01	2.27	3.75	[I, SST], IIN
Severe^b^	9 (7)	71.6 ± 7.7	-12.8	9.36	15.37	4.67	[I, IIT], SST

*Abbreviations*: *SAP* standard automated perimetry, *OCT* optical coherence tomography, *SD* standard deviation, *HFA* Humphrey Field Analyzer, *MD* mean deviation, *PSD* pattern standard deviation, *RNFL* retinal nerve fibre layer, *SSN* superior superonasal, *NSN* nasal superonasal, *IIN* inferior inferonasal, *I* inferior, *IIT* inferior inferotemporal, *TST* temporal superotemporal, *SST* superior superotemporal

^a^RNFL clock-hour sectors most often below normal limits (in rank order, bracketed sectors have equivalent rankings)

^b^Classification for each subject based on whichever of their eyes has the lowest HFA MD value; Mild: both eyes MD ≥ -6 dB; Moderate, worst eye MD < -6 dB but ≥ -12 dB; Severe, worst eye MD < -12 dB

**Fig. 2:**
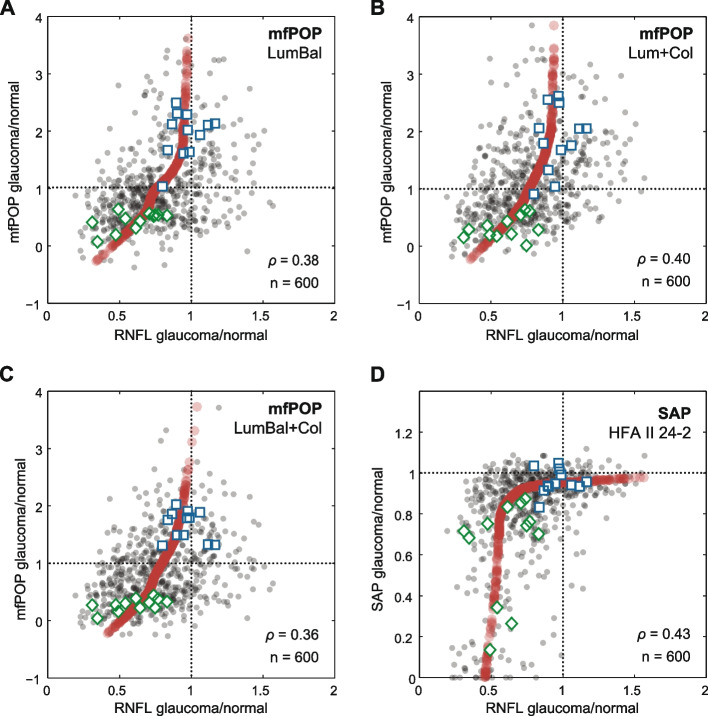
pRNFL sector/field test scatterplots and principal curves for all glaucoma eyes. Data points are plotted as semi-transparent (grey markers  ), therefore darker areas indicate higher concentrations of overlapping points. “*ρ*” (Spearman correlation coefficients) and “n” (number of points in the correlation) values shown at bottom right of each plot are across all glaucoma subjects. All correlations are significant at *p* < 0.0001. Also shown are the data for the glaucoma patient with the highest total correlations (  ,  ). Correlations for individual subjects varied between strong correlations in some, e.g. left (  ) and right (  ) eyes of the glaucoma patient shown (mfPOP LumBal: *ρ* = 0.84, Lum + Col: *ρ* = 0.81, LumBal + Col: *ρ* = 0.82; HFA: *ρ* = 0.80), to zero or negative correlations in others. As with the data points, principal curve outputs are shown as semi-transparent (red markers  ). These reveal non-linearities in the relationship between pRNFL and field sensitivities, this being most pronounced in SAP

**Fig. 3:**
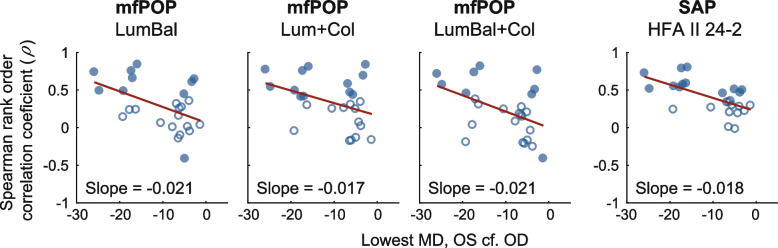
Correlation by MD in glaucoma. Spearman rank-order correlation coefficients for each glaucoma subject plotted against the MD of each subject’s worst-performing eye. Filled circles indicate that the correlation is significant at *p* < 0.05

**Fig. 4:**
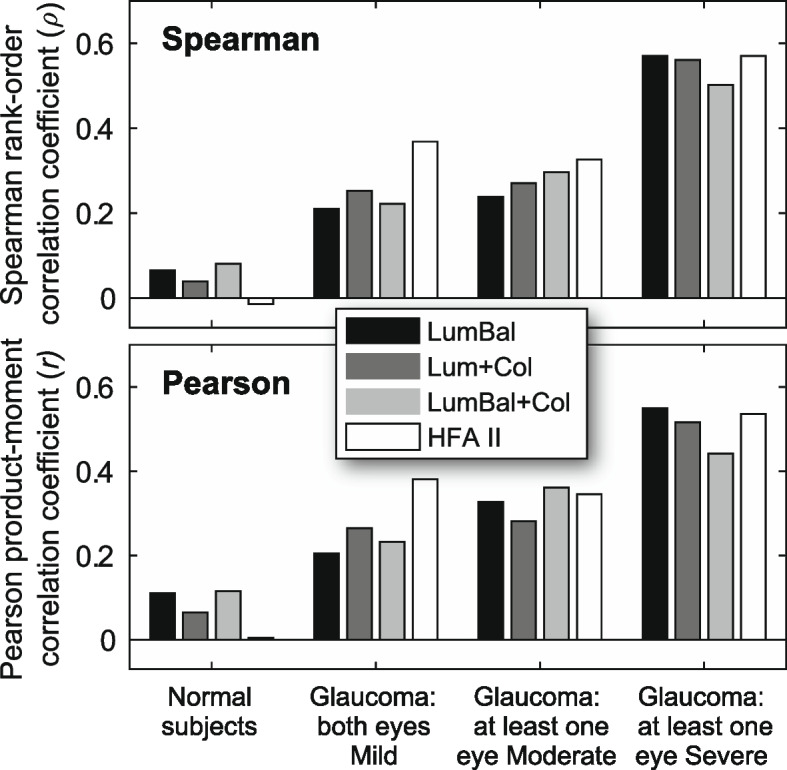
Spearman rho vs Pearson r comparison. Correlation coefficients for Spearman rank-order (*ρ*) and Pearson product-moment (*r*) correlation between SAP and mfPOP arcuate clusters, and RNFL sector thickness, by subject classification and test type

Positive correlations between pRNFL and pooled arcuate division mfPOP/SAP deviation scores are apparent in the scatterplots of data from all glaucoma subjects (Fig. 2). The principal curves (red dots) shown on these scatterplots reveal a much more linear relationship between the mfPOP and RNFL deviations than that of HFA. Plots of each subject’s Spearman correlation coefficients (*ρ*) against the MD of their worst-performing eye revealed similar slopes for both mfPOP and HFA tests, with all tests producing stronger correlations in subjects with lower function i.e. more advanced disease (Fig. 3). pRNFL deviations increased systematically with glaucoma severity in each of the mfPOP variants; SAP was slightly less consistent with a stronger correlation for patients with Mild eyes compared to those with at least one eye classified as Moderate (Fig. 4). No consistent differences were seen between mfPOP and SAP across glaucoma subgroups; SAP produced a stronger correlation for Mild eyes whereas mfPOP was somewhat stronger in normal subjects. Since these correlation coefficients represent measurements from a number of subjects they will likely be affected by inter-subject variability in measures of both structure and function. For the bulk of these analyses, Spearman correlation was chosen over Pearson due to the non-linear relationship between SAP and pRNFL thickness (Fig. 2D and [22]). Figure 4 (lower) shows that when Pearson correlation was used this somewhat favored mfPOP over HFA.

**Fig. 5:**
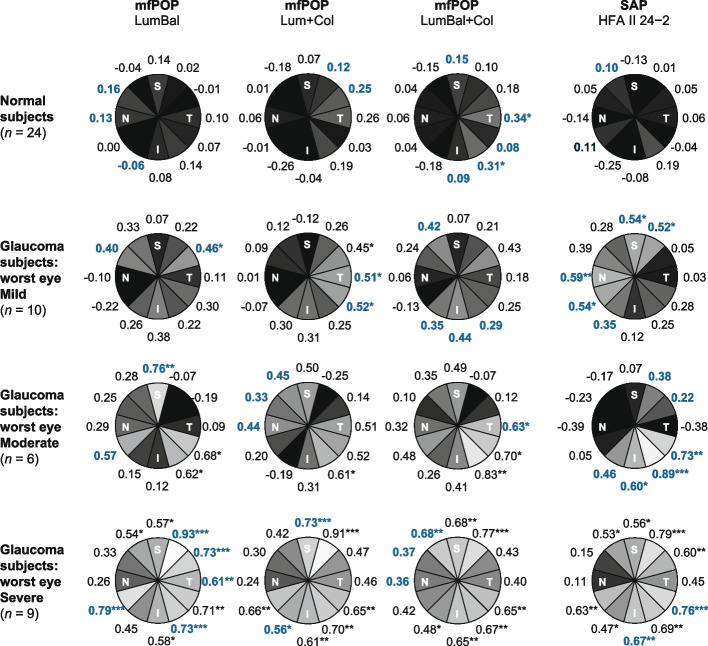
Correlation coefficients (*ρ*) by sector between SAP and mfPOP arcuate clusters, and RNFL sector thicknesses. Black shading represents zero or negative correlations; shading is lighter for higher correlations with white representing 100% correlation. Coefficients in bold/blue indicate the highest r of each subject classification for that sector across the different functional tests, i.e. across each row. Sectoral correlations were strongest in the superior superotemporal sector in patients with one eye classified Severe. The “*n*” reported below the subject subgroup titles represents the number of subjects in each subgroup. *P*-value degrees of freedom for Normal subjects and those with Mild, Moderate and Severe glaucoma are 46, 18, 10 and 16 respectively. (* *p* < 0.05, ** *p* < 0.01, *** *p* < 0.001). RNFL clock-hour sectors: S = superior, N = nasal, I = inferior, T = temporal

To establish which of the pRNFL clock-hour sectors were associated most strongly with the functional measures, correlations between OCT sector deviations and those of the pooled test-regions/point were performed by pRNFL clock-hour sector (Fig. 5). Although these results were somewhat variable, some clear patterns did emerge. Correlations in normal subjects were weak and mostly non-significant, particularly for SAP. Those of Mild glaucoma subjects were a little stronger, with SAP having the highest value in the largest number of sectors. The strongest structure–function relationships were observed in glaucoma subjects in the superotemporal sectors, e.g. mfPOP LumBal 0.93, Lum + Col 0.91 (Severe group), and in the inferotemporal sectors, SAP 0.89 (Moderate). The sectors with the strongest correlations were also outside normal OCT pRNFL limits in the largest number of subjects (Table 1, far-right column).

Sectoral correlations in the Moderate glaucoma group were, in general, between those of Mild and Severe, however null or negative correlations were present in each of the tests in some sectors in which a strong relationship would be expected e.g., superior and temporal sectors in SAP and superotemporal in mfPOP. The small size of the Moderate subset may have contributed to these results. In Severe glaucoma subjects the mfPOP LumBal test far outperformed the other mfPOP tests as well as SAP.

**Fig. 6:**
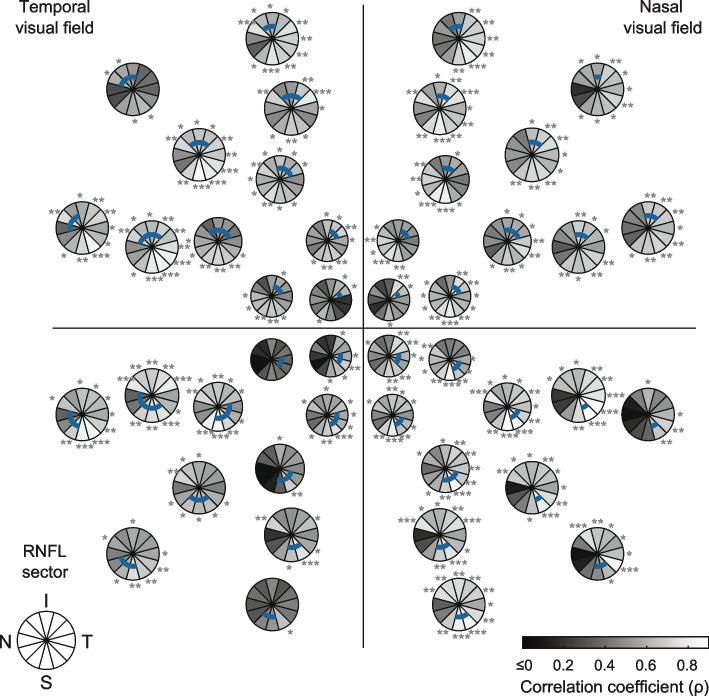
MfPOP / RNFL correlation coefficients (*ρ*) by mfPOP test-region for the LumBal test. Correlations are estimated across both eyes of glaucoma patients with one eye Severe. Blue arcs within each sector plot represent the RNFL sectors expected to correspond with that region of visual field. Range of color-bar does not reflect actual range of *ρ* values (which is -0.293 to 0.883): all values less than or equal to zero are shown in black. RNFL clock-hour sectors: S = superior, N = nasal, I = inferior, T = temporal. *P*-values for each correlation coefficient are indicated as follows: * *p* < 0.05, ** *p* < 0.01, *** *p* < 0.001

Correlation coefficients were also estimated between individual, rather than pooled, mfPOP test-region/HFA test-point deviations and each of the pRNFL sectors. These are shown in Figs. 6 (mfPOP) and 7 (SAP). This analysis provided an insight into the integrity of the sectoral divisions and showed which visual-field locations were most strongly correlated with their corresponding pRNFL sector. MfPOP LumBal test-regions and HFA test-points in congruent visual field locations produced correlations that were strongest in the same pRNFL sector over much of the visual field, although this was sometimes in a sector adjacent to that expected e.g. both LumBal test-regions and HFA test-points in the inferior field surrounding the midline produced strongest correlations in the superior-superotemporal sector, however in some of these regions the expected pRNFL sector was superior. Correlations tended to be stronger and more in accord with the expected pRNFL sector in more eccentric and more nasally-located visual field test-regions.

## Discussion

While the majority of variation in perimetric visual-field sensitivities is due to the number of functioning ganglion cells [24], the efficacy of perimetry in glaucoma is still largely reliant on the ability of the patient to accurately complete the procedure. Patients in poor health may find longer tests, or those requiring repeated responses, quite difficult to complete. In addition, the higher presence of media opacities in older patients make the use of tests utilizing short-wavelength light, or stimuli containing high spatial frequencies, problematic [25]. For these reasons, a test that can measure visual function quickly and objectively using longer-wavelength light, such as mfPOP, should have advantages in glaucoma assessment. Contradictory findings in rodent glaucoma models have led to some uncertainty regarding the relative effect of glaucoma on steady-state pupillary responses [26–28]. The use in mfPOP, however, of transient pupil responses to very short duration impulse stimuli, understood to be the result of visual signal mediated by ganglion cells and interneurons of the main visual pathway [10] and mediated through the extrastriate cortex [12, 15] allows us to objectively measure responses which should be affected by disease in a similar way to a patient’s visual percept. This study lends support to this hypothesis, with mfPOP producing comparable structure–function correlations to those derived using HFA.

Prior to this study, published findings of the relationship between pRNFL thickness and the pupillary response have largely been limited to investigations using ganzfeld stimuli and inter-eye asymmetry (see e.g. [29–31]). The only published report in which comparisons are made between the pRNFL and *focal* pupil stimuli failed to identify significant correlations [32]. Part of the reason for this may relate to a recent finding by our group that pupillary responses arising from the melanopsin-mediated ipRGC signal appear to be impaired in their ability to resolve glaucomatous scotomas [10]. The pretectal olivary nuclei of the pupillary pathway receive two distinct types of visual signal: ipRGC-mediated direct input which dominates the response to long duration stimuli, and input from various regions of visual cortex involved in the processing of conscious vision [12]. It is this latter cortical input that is understood to mediate pupillary responses to transient stimuli such as used in mfPOP [10, 15]. This divergent neural circuitry also leads to profound differences in the inferences that can be made using long-duration as opposed to transient stimuli. Steady-state ipRGC-mediated responses can only provide an indirect estimate of RGC-related visual loss since ipRGCs are not known to be involved in conscious vision in primates. This study thus provides the first evidence of a relationship between structural changes in the pRNFL and the amplitude of pupillary responses to stimulation of discrete areas of retina. Given our previous findings, this is not a result that would be expected from stimuli targeting very slow ipRGCs, with their low spatial-resolution [10].

**Fig. 7:**
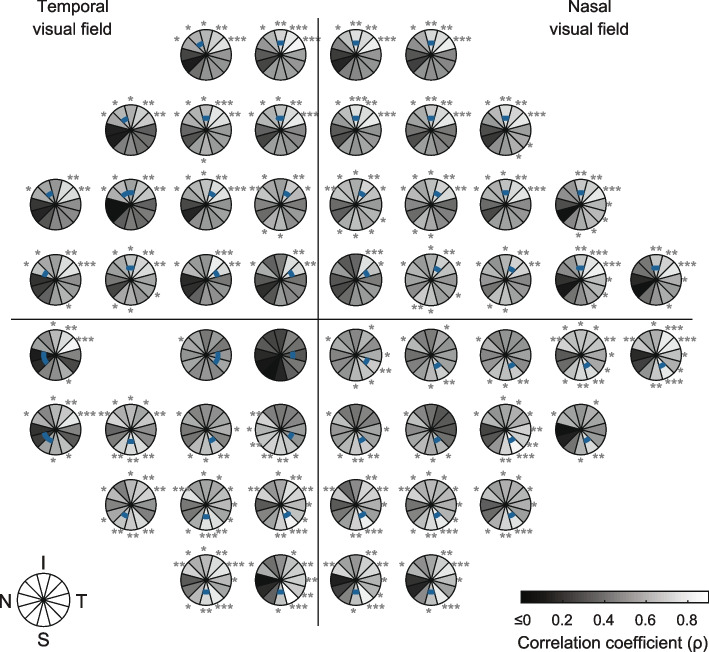
SAP / RNFL correlation coefficients (*ρ*) by SAP test-point location. Correlations are estimated across both eyes of glaucoma patients with one eye Severe. Blue arcs within each sector plot represent the RNFL sectors expected to correspond with that test-point location in the visual field. RNFL clock-hour sectors: S = superior, N = nasal, I = inferior, T = temporal. Range of values is -0.080 to 0.854: all values less than or equal to zero are shown in black. *P*-values for each correlation coefficient are indicated as follows: * *p* < 0.05, ** *p* < 0.01, *** *p* < 0.001

The large size of the mfPOP test-regions and extent of retina assessed using mfPOP is in stark contrast to the limited coverage of the 0.43° Goldmann size III stimuli used in HFA (Fig. 1). The effect of this is apparent in Figs. 6 and 7 where correlations were performed for each mfPOP test-region and HFA point. In SAP, mostly only single pRNFL sectors originated at each test-point, however for mfPOP test-regions this ranged between 1 and 5. It might have been expected then that the correlation between mfPOP test-regions and each individual pRNFL sector would be lower than SAP due to more than one clock-hour sector having axons originating in that test-region. However, this is not the case in the majority of regions, where mfPOP correlations in sectors where damage would be expected according to axon trajectories (blue arcs, Figs. 6 and 7) are equivalent to, and, in some cases, stronger than the single sectors of HFA test-points. This effect could potentially have been due to correlation in pRNFL damage between adjacent arcuate divisions of retina; however, this would have produced a similar pattern in the adjacent non-corresponding sectors of HFA test-regions, which is not apparent. One interpretation of this is that the large extent of mfPOP test-regions gives access to additional, rather than averaged, information regarding function. This is perhaps not unexpected, given the difference between the proportion of retina assessed using the two methods. The use of Goldmann size V stimuli, or SITA Standard or Full-Threshold strategy rather than SITA FAST, could potentially have provided stronger results for SAP, however the difference in test–retest variability between these strategies is only a fraction of the extent of the overall variability of either method [33], so any improvement would be expected to be minimal.

As might be anticipated, and as is reflected in studies of the diagnostic utility of the pupillary response in glaucoma [8, 9, 34], the strongest correlations were observed in the Severe subset of subjects. Although the greatest magnitude mfPOP correlations in Severe glaucoma were produced using the LumBal test, in Mild and Moderate disease overall structure–function correlations (Fig. 3) were strongest in the mfPOP tests which incorporated color-signal as well as luminance. This pattern mirrors the higher sensitivity and specificity we have previously observed using this type of composite stimulus in early glaucoma [8] adding conviction to the idea that the addition of color information in the Lum + Col and LumBal + Col stimuli has advantages in early disease.

The relatively small number of subjects in each of the glaucoma subgroups is a likely source of variability in this study. Perhaps the best example of this can be seen in the sectoral correlations shown in Fig. 4. In the six glaucoma subjects classified as Moderate, zero and negative SAP correlations in superonasal sectors were accompanied by positive mfPOP correlations; in the same subjects, zero and negative mfPOP correlations in the superior superotemporal sector accompanied a positive SAP correlation. In contrast to this variability, however, is the presence of clear and consistent strengthening of inferotemporal correlations between the Mild and Moderate groups. Inaccuracies in the mapping of clock-hour sectors to retinal locations and inter-individual differences in axon trajectories will also have introduced variability into this analysis. The use of individualized retinal maps incorporating simple parameters like optic nerve head position and axial length, such as proposed by Denniss et al. [35], would likely produce more accurate correlations than those obtained using population averages as we have in this study. However, the aims of this project were to compare SAP structure–function relationships with those of mfPOP, and different mfPOP methods with each other, rather than to provide absolute estimates of the degree of correlation, so given that both test types were subject to the same constraints, this should not affect the conclusions drawn from this data.

## Conclusions

The strong structure–function relationship between pRNFL thickness and mfPOP response amplitudes in Severe disease, consistently better than that of SAP, makes a convincing argument for the mfPOP method’s further development and utility in late-stage disease. Recent developments in the mfPOP method, so called clustered volleys presentation, have improved signal to noise ratios by 40% compared to the methods used here [1]. Thus further improvements in structure–function correlation may be possible for mfPOP. The relatively linear scatterplots of Fig. 2 and the predominantly positive correlations, even in eyes of normal subjects, suggest that mfPOP tracks pRNFL thickness relatively well. In summary, this study has further demonstrated the viability of mfPOP as an emerging perimetric method and lent further evidence to the contribution of neurons in the main visual pathway to mfPOP responses.

### Supplementary Information

**Supplementary Material 1.**

**Supplementary Material 2.**

**Supplementary Material 3.**

## Data Availability

The datasets used and/or analysed during the current study are available from the corresponding author on reasonable request.

## Abbreviations

HFA: Humphrey Field Analyzer
ipRGC: Intrinsically photosensitive retinal ganglion cell
MD: Mean deviation
mfPOP: Multifocal pupillographic objective perimetry
OCT: Optical coherence tomography
pRNFL: Peripapillary retinal nerve fibre layer
PSD: Pattern standard deviation
RGC: Retinal ganglion cell
RNFL: Retinal nerve fibre layer
SAP: Standard automated perimetry
