# Supplementary material for: The structure–function relationship between multifocal pupil perimetry and retinal nerve fibre layer in glaucoma
Source: BMC Ophthalmol. 2024 Apr 10;24:159. doi: 10.1186/s12886-024-03402-z (PMC11008001; doi:10.1186/s12886-024-03402-z)
Supplement: Supplementary file 1 — Supplementary Material 1. [file 12886_2024_3402_MOESM1_ESM.pdf]

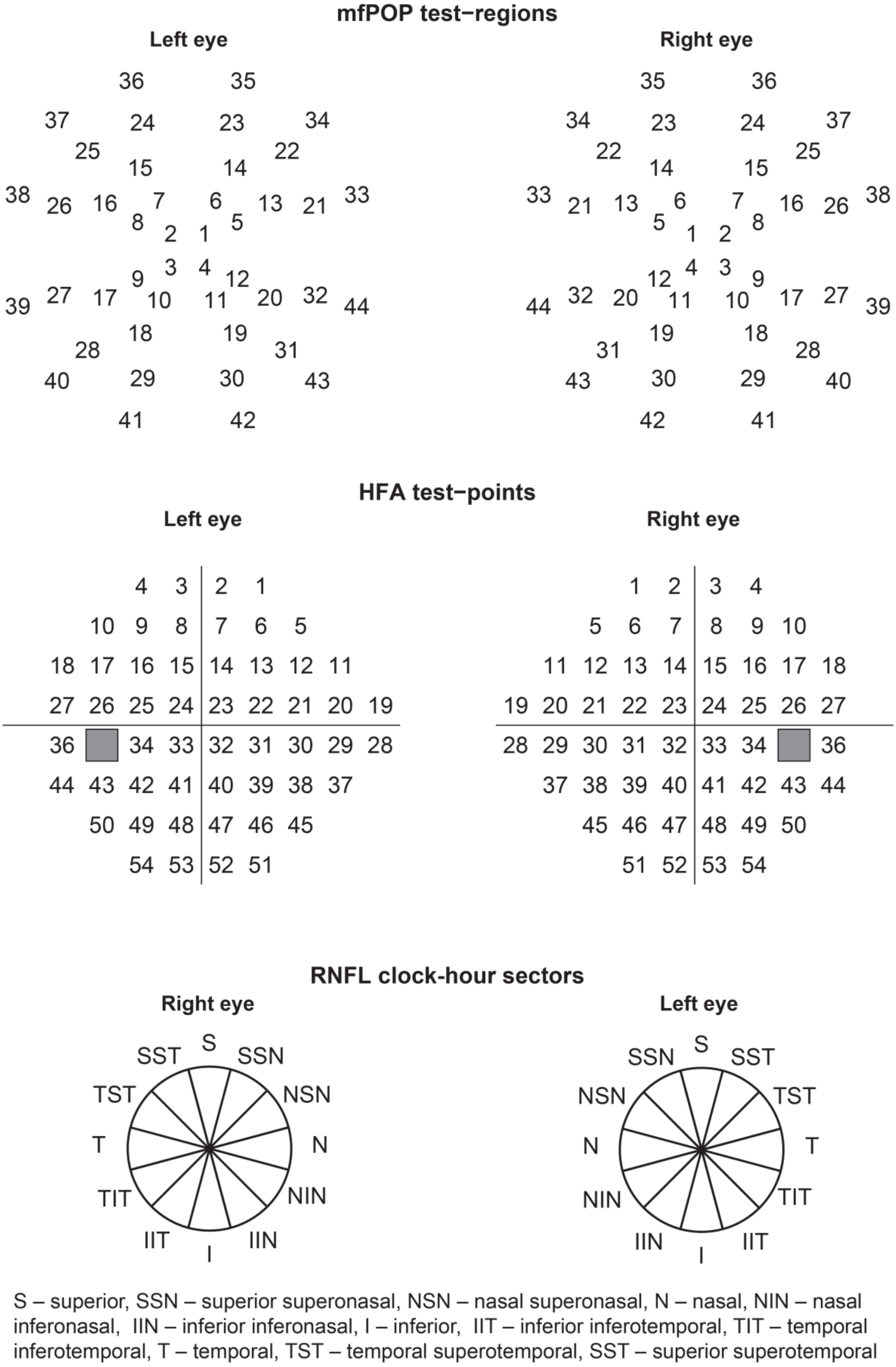

Supplementary Figure S1. Numbering of mfPOP test-regions and HFA test-points, and labelling of RNFL clock-hour sectors used in Supplementary Tables S1 and S2.
