# Supplementary material for: The structure–function relationship between multifocal pupil perimetry and retinal nerve fibre layer in glaucoma
Source: BMC Ophthalmol. 2024 Apr 10;24:159. doi: 10.1186/s12886-024-03402-z (PMC11008001; doi:10.1186/s12886-024-03402-z)
Supplement: Supplementary file 2 — Supplementary Material 2. [file 12886_2024_3402_MOESM2_ESM.pdf]

**Supplementary Table S1.** Weighting of mfPOP test-regions according to their contribution to retinal arcuate divisions corresponding to Stratus OCT sectors.

| MfPOP test-region* | Test-region ring: central→ peripheral | RNFL clock-hour sector* | | | | | | | | | | | |
|---|---|---|---|---|---|---|---|---|---|---|---|---|---|
| | | S | SSN | NSN | N | NIN | IIN | I | IIT | TIT | T | TST | SST |
| **1** | | 0 | 0 | 0 | 0 | 0 | 0 | 0 | 0 | 1 | 0 | 0 | 0 |
| **2** | 1 | 0 | 0 | 0 | 0 | 0 | 0 | 0 | 0 | 1 | 0 | 0 | 0 |
| **3** | | 0 | 0 | 0 | 0 | 0 | 0 | 0 | 0 | 0 | 0.75 | 0.25 | 0 |
| **4** | | 0 | 0 | 0 | 0 | 0 | 0 | 0 | 0 | 0 | 0.25 | 0.75 | 0 |
| **5** | | 0 | 0 | 0 | 0 | 0 | 0 | 0 | 0.66 | 0.34 | 0 | 0 | 0 |
| **6** | | 0 | 0 | 0 | 0 | 0 | 0 | 0 | 0.8 | 0.2 | 0 | 0 | 0 |
| **7** | | 0 | 0 | 0 | 0 | 0 | 0 | 0 | 0.75 | 0.25 | 0 | 0 | 0 |
| **8** | 2 | 0 | 0 | 0 | 0 | 0 | 0 | 0 | 0.25 | 0.75 | 0 | 0 | 0 |
| **9** | | 0 | 0 | 0 | 0 | 0 | 0 | 0 | 0 | 0 | 0.5 | 0.5 | 0 |
| **10** | | 0 | 0 | 0 | 0 | 0 | 0 | 0 | 0 | 0 | 0.05 | 0.7 | 0.25 |
| **11** | | 0 | 0 | 0 | 0 | 0 | 0 | 0 | 0 | 0 | 0 | 0.4 | 0.6 |
| **12** | | 0 | 0 | 0 | 0 | 0 | 0 | 0 | 0 | 0 | 0 | 0.35 | 0.65 |
| **13** | | 0 | 0 | 0 | 0 | 0 | 0 | 0.1 | 0.8 | 0.1 | 0 | 0 | 0 |
| **14** | | 0 | 0 | 0 | 0 | 0 | 0 | 0.4 | 0.6 | 0 | 0 | 0 | 0 |
| **15** | | 0 | 0 | 0 | 0 | 0 | 0 | 0.45 | 0.5 | 0.05 | 0 | 0 | 0 |
| **16** | 3 | 0 | 0 | 0 | 0 | 0 | 0.1 | 0.3 | 0.25 | 0.35 | 0 | 0 | 0 |
| **17** | | 0.2 | 0 | 0 | 0 | 0 | 0 | 0 | 0 | 0 | 0.3 | 0.3 | 0.2 |
| **18** | | 0.3 | 0 | 0 | 0 | 0 | 0 | 0 | 0 | 0 | 0 | 0.3 | 0.4 |
| **19** | | 0.05 | 0 | 0 | 0 | 0 | 0 | 0 | 0 | 0 | 0 | 0.15 | 0.8 |
| **20** | | 0 | 0 | 0 | 0 | 0 | 0 | 0 | 0 | 0 | 0 | 0.1 | 0.9 |
| **21** | | 0 | 0 | 0 | 0 | 0 | 0 | 0.45 | 0.55 | 0 | 0 | 0 | 0 |
| **22** | | 0 | 0 | 0 | 0 | 0 | 0 | 0.75 | 0.25 | 0 | 0 | 0 | 0 |
| **23** | | 0 | 0 | 0 | 0 | 0 | 0 | 0.8 | 0.2 | 0 | 0 | 0 | 0 |
| **24** | | 0 | 0 | 0 | 0 | 0 | 0.1 | 0.8 | 0.1 | 0 | 0 | 0 | 0 |
| **25** | | 0 | 0 | 0 | 0 | 0 | 0.5 | 0.4 | 0.1 | 0 | 0 | 0 | 0 |
| **26** | 4 | 0 | 0 | 0 | 0 | 0.3 | 0.4 | 0.2 | 0.1 | 0 | 0 | 0 | 0 |
| **27** | | 0.15 | 0.3 | 0.2 | 0.25 | 0 | 0 | 0 | 0 | 0 | 0 | 0 | 0.1 |
| **28** | | 0.6 | 0.25 | 0 | 0 | 0 | 0 | 0 | 0 | 0 | 0 | 0 | 0.15 |
| **29** | | 0.75 | 0 | 0 | 0 | 0 | 0 | 0 | 0 | 0 | 0 | 0 | 0.25 |
| **30** | | 0.4 | 0 | 0 | 0 | 0 | 0 | 0 | 0 | 0 | 0 | 0 | 0.6 |
| **31** | | 0 | 0 | 0 | 0 | 0 | 0 | 0 | 0 | 0 | 0 | 0 | 1 |
| **32** | | 0 | 0 | 0 | 0 | 0 | 0 | 0 | 0 | 0 | 0 | 0 | 1 |
| **33** | | 0 | 0 | 0 | 0 | 0 | 0 | 0.9 | 0.1 | 0 | 0 | 0 | 0 |
| **34** | | 0 | 0 | 0 | 0 | 0 | 0 | 1 | 0 | 0 | 0 | 0 | 0 |
| **35** | | 0 | 0 | 0 | 0 | 0 | 0.1 | 0.9 | 0 | 0 | 0 | 0 | 0 |
| **36** | | 0 | 0 | 0 | 0 | 0 | 0.5 | 0.5 | 0 | 0 | 0 | 0 | 0 |
| **37** | | 0 | 0 | 0 | 0 | 0.1 | 0.8 | 0.1 | 0 | 0 | 0 | 0 | 0 |
| **38** | 5 | 0 | 0 | 0 | 0.1 | 0.7 | 0.2 | 0 | 0 | 0 | 0 | 0 | 0 |
| **39** | | 0 | 0.2 | 0.45 | 0.35 | 0 | 0 | 0 | 0 | 0 | 0 | 0 | 0 |
| **40** | | 0.2 | 0.7 | 0.1 | 0 | 0 | 0 | 0 | 0 | 0 | 0 | 0 | 0 |
| **41** | | 0.8 | 0.2 | 0 | 0 | 0 | 0 | 0 | 0 | 0 | 0 | 0 | 0 |
| **42** | | 0.85 | 0 | 0 | 0 | 0 | 0 | 0 | 0 | 0 | 0 | 0 | 0.15 |
| **43** | | 0.2 | 0 | 0 | 0 | 0 | 0 | 0 | 0 | 0 | 0 | 0 | 0.8 |
| **44** | | 0 | 0 | 0 | 0 | 0 | 0 | 0 | 0 | 0 | 0 | 0 | 1 |

*Refer Supplementary Figure S1 for test-region and RNFL sector locations.

**Abbreviations:** RNFL - retinal nerve fiber layer, S – superior, SSN – superior superonasal, NSN – nasal superonasal, N – nasal, NIN – nasal inferonasal, IIN – inferior inferonasal, I – inferior, IIT – inferior inferotemporal, TIT – temporal inferotemporal, T – temporal, TST – temporal superotemporal, SST – superior superotemporal
