# Supplementary material for: The structure–function relationship between multifocal pupil perimetry and retinal nerve fibre layer in glaucoma
Source: BMC Ophthalmol. 2024 Apr 10;24:159. doi: 10.1186/s12886-024-03402-z (PMC11008001; doi:10.1186/s12886-024-03402-z)
Supplement: Supplementary file 3 — Supplementary Material 3. [file 12886_2024_3402_MOESM3_ESM.pdf]

Supplementary Table S2. Weighting of HFA test points according to their contribution to retinal arcuate divisions corresponding to Stratus OCT sectors.

HFA test-point*	Row S → I	Column N → T	RNFL clock-hour sector*											
			S	SSN	NSN	N	NIN	IIN	I	IIT	TIT	T	TST	SST
**1**		4	0	0	0	0	0	0	1	0	0	0	0	0
**2**	1	5	0	0	0	0	0	0	1	0	0	0	0	0
**3**		6	0	0	0	0	0	0	1	0	0	0	0	0
**4**		7	0	0	0	0	0	1	0	0	0	0	0	0
**5**		3	0	0	0	0	0	0	1	0	0	0	0	0
**6**		4	0	0	0	0	0	0	1	0	0	0	0	0
**7**	2	5	0	0	0	0	0	0	1	0	0	0	0	0
**8**		6	0	0	0	0	0	0	1	0	0	0	0	0
**9**		7	0	0	0	0	0	0	1	0	0	0	0	0
**10**		8	0	0	0	0	0	1	0	0	0	0	0	0
**11**		2	0	0	0	0	0	0	1	0	0	0	0	0
**12**		3	0	0	0	0	0	0	1	0	0	0	0	0
**13**		4	0	0	0	0	0	0	0	1	0	0	0	0
**14**	3	5	0	0	0	0	0	0	0	1	0	0	0	0
**15**		6	0	0	0	0	0	0	0	1	0	0	0	0
**16**		7	0	0	0	0	0	0	0	1	0	0	0	0
**17**		8	0	0	0	0	0	0.25	0.75	0	0	0	0	0
**18**		9	0	0	0	0	0	1	0	0	0	0	0	0
**19**		1	0	0	0	0	0	0	1	0	0	0	0	0
**20**		2	0	0	0	0	0	0	1	0	0	0	0	0
**21**		3	0	0	0	0	0	0	0	1	0	0	0	0
**22**		4	0	0	0	0	0	0	0	1	0	0	0	0
**23**	4	5	0	0	0	0	0	0	0	0	1	0	0	0
**24**		6	0	0	0	0	0	0	0	0	1	0	0	0
**25**		7	0	0	0	0	0	0	0	0	1	0	0	0
**26**		8	0	0	0	0	0	0	1	0	0	0	0	0
**27**		9	0	0	0	0	1	0	0	0	0	0	0	0
**28**		1	0	0	0	0	0	0	0	0	0	0	0	1
**29**		2	0	0	0	0	0	0	0	0	0	0	0	1
**30**		3	0	0	0	0	0	0	0	0	0	0	0	1
**31**		4	0	0	0	0	0	0	0	0	0	0	0	1
**32**	5	5	0	0	0	0	0	0	0	0	0	0	1	0
**33**		6	0	0	0	0	0	0	0	0	0	1	0	0
**34**		7	0	0	0	0	0	0	0	0	0	0.75	0.25	0
**35**		8	- - - - - - - - Blind spot (ONH)- - - - - - - -											
**36**		9	0	0	0.25	0.75	0	0	0	0	0	0	0	0
**37**		2	0	0	0	0	0	0	0	0	0	0	0	1
**38**		3	0	0	0	0	0	0	0	0	0	0	0	1
**39**		4	0	0	0	0	0	0	0	0	0	0	0	1
**40**	6	5	0	0	0	0	0	0	0	0	0	0	0	1
**41**		6	0	0	0	0	0	0	0	0	0	0	1	0
**42**		7	0	0	0	0	0	0	0	0	0	0	0	1
**43**		8	1	0	0	0	0	0	0	0	0	0	0	0
**44**		9	0	0.5	0.5	0	0	0	0	0	0	0	0	0
**45**		3	0	0	0	0	0	0	0	0	0	0	0	1
**46**		4	0	0	0	0	0	0	0	0	0	0	0	1
**47**	7	5	0	0	0	0	0	0	0	0	0	0	0	1
**48**		6	0	0	0	0	0	0	0	0	0	0	0	1
**49**		7	1	0	0	0	0	0	0	0	0	0	0	0
**50**		8	0	1	0	0	0	0	0	0	0	0	0	0
**51**		4	1	0	0	0	0	0	0	0	0	0	0	0
**52**	8	5	1	0	0	0	0	0	0	0	0	0	0	0
**53**		6	1	0	0	0	0	0	0	0	0	0	0	0
**54**		7	1	0	0	0	0	0	0	0	0	0	0	0

*Refer Supplementary Figure S1 for test-point and RNFL sector locations.

Abbreviations: RNFL - retinal nerve fiber layer, S – superior, SSN – superior superonasal, NSN – nasal superonasal, N – nasal, NIN – nasal inferonasal, IIN – inferior inferonasal, I – inferior, IIT – inferior inferotemporal, TIT – temporal inferotemporal, T – temporal, TST – temporal superotemporal, SST – superior superotemporal
